# Climate Change and Reproductive Health

**DOI:** 10.1210/endrev/bnaf026

**Published:** 2025-08-04

**Authors:** Anna Claire G Fernandez, Sanika Pelnekar, Joshua F Robinson, Gary M Shaw, Amy M Padula, Tracey J Woodruff, Linda C Giudice

**Affiliations:** Department of Emergency Medicine, University of California San Francisco, San Francisco, CA 94143, USA; International Federation of Fertility Societies, Houston, TX 77043, USA; Department of Obstetrics, Gynecology and Reproductive Sciences, University of California San Francisco, San Francisco, CA 94143, USA; Department of Pediatrics, Stanford University, Stanford, CA 94304, USA; Department of Obstetrics, Gynecology and Reproductive Sciences, University of California San Francisco, San Francisco, CA 94143, USA; Department of Obstetrics, Gynecology and Reproductive Sciences, University of California San Francisco, San Francisco, CA 94143, USA; Department of Obstetrics, Gynecology and Reproductive Sciences, University of California San Francisco, San Francisco, CA 94143, USA

**Keywords:** climate change, reproductive health, pregnancy, pollution, endocrine-disrupting chemicals, extreme weather

## Abstract

Climate change is a major threat to the world's population and is due to global warming from human activities that increase atmospheric greenhouse gas levels—burning fossil fuels, industry emissions, vehicular exhaust, and aerosol chlorofluorocarbons—that trap heat in the Earth's atmosphere and adversely impact air quality. Resulting higher global temperatures, extreme weather events, and rising sea levels lead to greater frequency of wildfires and floods, which, in turn, result in population displacements and threaten air and water quality, food and water security, economic and public health infrastructures, and societal safety. Climate change has direct and indirect impacts on human health and well-being across the globe, with disproportionate impact on vulnerable populations including women, pregnant persons, the developing fetus, children, older adults, indigenous peoples, persons with disabilities, preexisting and/or chronic medical conditions, and low income and communities of color. As consequences of climate change, global mortality and noncommunicable diseases are mounting because of lack of progress to reverse current trends. Climate change effects on reproductive processes and outcomes have received less attention globally, despite huge consequences for human development, fertility, and pregnancy outcomes. This review provides evidence for direct and indirect effects of climate change on human health with a focus on reproductive processes and outcomes based on experimental models and epidemiologic data, and strategies to mitigate harms. The goal is to increase awareness about climate effects on reproductive health among clinicians, researchers, the public, and policymakers, and to engage all stakeholders to change the current trajectory of harm.

## Essential Points

- The primary source of climate change is pollution from fossil fuel combustion and related industrial processes that pose risks to human reproductive health (infertility, compromised fetal development, poor pregnancy outcomes) with consequences across the lifespan
- Climate change effects can be direct and indirect, and social determinants of health can mediate these exposures, widening health disparities in low-resource populations
- Climate change-driven weather events and wildfires contribute to the redistribution and increase in air pollutants (eg, fine particulate matter), endocrine disrupting chemicals and other contaminants in food, water and soil, leading to heightened human exposure
- Educating the public, clinicians, researchers, and health policy makers is essential to change the course of climate change for this and future generations

Climate change is driven by increasing levels of atmospheric greenhouse gasses, including carbon dioxide, methane, nitrous oxide, and chlorofluorocarbons. These gases are released through the burning of fossil fuels, industrial emissions, vehicular exhaust, and the use of aerosol products containing chlorofluorocarbons (1, 2). Greenhouse gasses create a heat-trapping layer in the atmosphere, leading to global warming and disruption to the atmospheric and ecological balance, with widespread implications for global health (2). The consequences of this trend extend beyond rising temperatures, encompassing an array of environmental hazards such as deteriorating air quality and wildfires, alterations in the distribution of availability of toxic chemicals, extreme heat, increased severe weather events (3), and rising sea levels—all of which compound health risks and produce complex impacts on human well-being (4). Reproductive health has only recently been included in the risk paradigm for these events (5-8) and is the subject of this review.

Since the mid-18th century, anthropogenic activities contributing to climate change have increasingly impacted human health outcomes (9-16), especially as the rise in fossil fuel consumption now exceeds 15 times that of the 1950s (17). Climate change directly impacts health via multiple ecological avenues including excess heat, wildfires, hurricanes, and floods. The same primary source that contributes to climate change (ie, fossil fuel production) produces a host of primary environmental health risk factors. For example, air pollutants such as particulate matter (PM), nitrogen oxides, sulfur dioxide, and volatile organic compounds are emitted into the environment as byproducts of fossil fuel combustion for energy production, transportation, and industrial processes (1). Communities located near areas with increased exposure to traffic and industrial-related pollution are at increased disease risk, for example, cardiovascular and cerebrovascular disease incidence (18). Although the detrimental impacts of air pollution on pulmonary and cardiovascular disease incidence are well established, less attention has been given to its short- and long-term effects on reproductive health (9). In addition, climate change-induced events such as droughts, floods, and extreme weather alter the redistribution and bioavailability of many harmful chemicals including endocrine disrupting chemicals (EDCs), heavy metals, pesticides, and other pollutants, compounding the health risks.

Climate change significantly impacts reproductive health, affecting fertility, fetal development, and pregnancy outcomes, and with consequences that can manifest later in life, influencing adult health and disease susceptibility. Here, we review multifaceted effects of climate change on reproductive health, addressing both direct and indirect impacts stemming from extreme weather events, air pollution, and chemical exposures. We highlight how environmental factors influence reproductive outcomes and fertility via both direct and indirect mechanisms. Direct impacts on reproductive health consist of climate-caused exposures that alter physiology and biological processes, mediated largely through disrupted endocrine signaling and endocrine-immune interactions in the reproductive tissues and systemically (19-21), resulting from excess heat, wildfires, and extreme weather, although exact mechanisms are not always well understood. Indirect impacts, on the other hand, refer to downstream events that mediate exposure to contaminants, infections and stress, ultimately impacting reproductive health. Examples of these downstream phenomena include air pollution, EDCs, and vectors of infectious disease, which, although not a manifestation of direct extreme weather events themselves, play a pivotal role in reproductive outcomes. Notably, social determinants of health such as poverty and barriers to health care can mediate these exposures, widening health disparities in low-resource populations. This review is structured to examine hazardous exposures driven by climate change, their effects on reproductive health, disparities across communities, and mitigation strategies.

## Methodology

**Figure 1.**
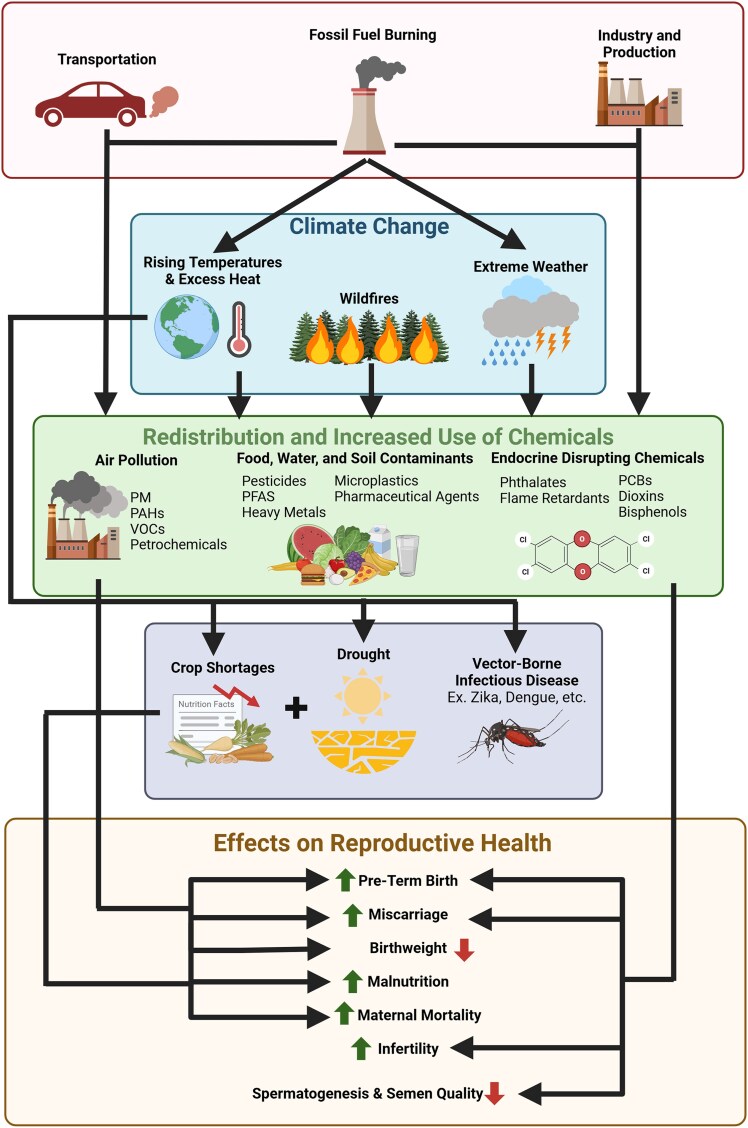
Summary of direct and indirect effects of climate change leading to downstream reproductive outcomes (22). Figure created in Biorender.

We conducted a review that is based on findings from authoritative bodies and select systematic reviews supplemented by narrative analysis to synthesize studies that examine reproductive and endocrine-mediated health impacts caused by climate change-driven environmental exposures. We address both direct and indirect impacts stemming from extreme weather events, air pollution, and chemical exposures. The review highlights how these environmental factors influence reproductive outcomes and fertility while also considering broader public health and social implications. The discussion is structured to examine each key area of impact systematically. First, it draws on findings from authoritative and systematic reviews, providing a foundation of established knowledge. Then, it delves deeper into additional scientific considerations, focusing on the biological mechanisms, when known, through which these environmental stressors may contribute to adverse reproductive health outcomes. Our goal is to provide a comprehensive understanding of the interplay between climate change and reproductive outcomes, while also proposing actionable strategies for mitigating these effects to protect public health in an increasingly vulnerable global environment. As depicted in Fig. 1, this review explores the holistic impact of climate change on reproductive health by examining both immediate and downstream impacts on endocrinology, pregnancy outcomes, and reproductive health. The strength of this approach is the presentation of multimodal biological and social mechanisms that influence reproductive and public health outcomes.

## Environmental Exposures in a Changing Climate and Reproductive Health (Direct Effects)

Climate change is fueled by pollution sources that also pose risks to human health, and the primary source of climate change is fossil fuel combustion and related industrial processes. Industrial and transport-related emissions create a feedback loop that increases the intensity of greenhouse gas emissions, creating excess heat, wildfires, and hurricanes and floods (23). The distribution of health impacts related to climate change is also not uniform: resource-poor populations are especially vulnerable and experience health disparities related to increased pollution exposures (24). In the United States, the relationship between poverty and exposure to air pollution can be traced back to historical redlining, where zoning laws and urban planning projects in the post-war era deliberately disadvantaged poor residents of color and increased exposures to environmental hazards (25).

### Excess Heat

Increasing temperatures are a hallmark of climate change and have impacts on human health directly (eg, extreme heat waves), and they instigate further weather patterns that in turn impact health (eg, extreme weather events, drought). Extreme heat poses several pregnancy and reproductive risks through osmotic, contaminant and stress-related mechanisms (26-28). Pregnancy naturally involves increased blood volume and higher hydration requirements, making pregnant individuals more susceptible to dehydration when exposed to high temperatures (27). Dehydration, in combination with heat stress, can disrupt blood flow to the placenta, potentially resulting in complications such as preterm uterine contractions, fetal growth restriction, and placental abruption (29). Additionally, elevated maternal core body temperature and pulse due to heat exposure can lead to fetal tachycardia and induce uterine contractions (26). Heat can also stimulate the release of antidiuretic hormones and oxytocin, which may further trigger uterine activity (26).

Extreme heat and other climate change-related phenomena show associations with adverse birth outcomes (30). Analysis of extreme heat exposures and increased risk of selected birth defects including heart defects, neural tube defects, and orofacial clefts have also underscored the correlation between heat and poor birth outcomes (31-36). Maternal exposure to elevated ambient temperatures, particularly later in pregnancy, are associated with preterm birth and stillbirth (11, 37, 38). Moreover, extreme heat is often associated with low birthweight, particularly in low- and middle- income countries, likely involving metabolic and physiologic insults, as well as socioeconomic factors, including limited accessed to cooled environments and water sources. Two unique reports found heat associated with increased rapid neonatal weight gain (38-40). Although the underlying mechanism is not known with certainty, it may involve brown adipose tissue—a key regulatory of neonatal thermoregulation and metabolism (39-41). Both short- and long-term heat stress is associated with stillbirth and spontaneous abortion with vulnerability in late pregnancy globally (37, 38, 42-45).

In general, inferences from research on heat and reproductive health are challenged by the fact that extreme heat exposure has been variably defined based on temperature (eg, mean, maximum, upper percentile), on duration at that temperature (eg, 2-7 days), and on the investigated timing of exposure during pregnancy. Mechanisms that may underlie an association between extreme heat and various adverse outcomes of pregnancy are not well understood, but are hypothesized to involve inflammation, maternal heat stress, dehydration, placental dysfunction, and increased oxidative stress (46, 47).

Pregnancy increases susceptibility to health risks and plays a critical role in shaping long-term health trajectories from physiologic changes such as increased respiratory rate and cardiac output (48). Conditions acquired in pregnancy can also provide a preview to future health risks; for example, gestational hypertension and gestational diabetes are associated with a higher risk of developing chronic hypertension and type 2 diabetes later in life, respectively (49). Additionally, delivering a low birthweight infant has been linked to increased risk of future maternal cardiovascular disease (50). Gestational hypertension can complicate up to 10% of pregnancies, occurring as maternal blood volume and cardiac output increase to support the growth and development of the fetus (51). Both heat stress and air pollution exacerbate the risk of hypertension in pregnancy, through complex and interactive mechanisms (52), compounded by social determinants of health, such as poverty (52).

The combined impact of heat and air pollution are of particular concern, as they often co-occur and cause interactions that increase levels of pollutants such as ozone and volatile organic compounds. Although some effort has been made to explore possible modifying influences between extreme heat and air pollutants (53), more research would be needed in this area. Interestingly, extreme heat exposure and preterm birth risk has recently been observed to be positively influenced by level of green spaces, such as parks and natural recreation areas, and more affluent socioeconomic status (54). Proximity to greenness and associated reductions in risk is a growing area of inquiry in studies of perinatal outcomes (55). The attendant reasoning for the reduction includes lower temperatures and fewer pollutants associated with proximity to greenspace.

In addition to effects on pregnancy, increasing ambient temperatures correlate with poor sperm and semen parameters, affecting male reproductive function. Specifically, reduced sperm motility and greater DNA fragmentation have been reported (56). Male reproductive health and testosterone biosynthesis in Leydig cells decreases in response to heat stress, potentially mediated by the repression of the steroidogenic acute regulatory protein (57). Effects on female gametogenesis is less well studied, and impacts on reproductive competence is worthy of further investigation as extreme weather events continue to rise.

#### Wildfires

Wildfires have quadrupled in size and intensity over the past 40 years across the United States (58), catalyzed by warmer, drier conditions and increased incidences of drought (59). The smoke emitted by these wildfires comprises 25% of PM_2.5_ found in American air pollution, with the Western United States bearing the greatest burden of these emissions (60, 61). Exposure to wildfire smoke can increase the risk of respiratory conditions, such as asthma and exacerbation of chronic obstructive pulmonary disease (62), heart disease, and neurological conditions.

Most studies on this topic are heterogeneous with respect to almost every aspect of study design, including exposure assessment, outcome ascertainment, confounding adjustment, level of observation, and statistical methods. Several studies have reported associations between exposure during pregnancy and preterm birth (birth <37 weeks' gestation) with evidence from numerous locations in the United States and globally (37, 46). Despite various methods of exposure assessment, results were remarkably consistent and indicated an increased risk of preterm birth (63-65). Interestingly, investigations of birthweight associations with wildfires have been more equivocal (66-71).

Investigations of wildfire smoke on other pregnancy outcomes are fewer. Wildfire exposure has been associated with stillbirth and pregnancy loss (ie, miscarriage) in nonhuman primates in California (72, 73). A few studies with varied outcome ascertainment and exposure assessment have suggested associations between wildfire smoke and various birth defects including spina bifida (74) and gastroschisis (75), but each finding has yet to be confirmed in subsequent studies. Two studies examined the impact of wildfire smoke on sex ratio. One found a decrease in male births in Australia, whereas the other found no difference in California (65, 71). Associations have also been observed between wildfire smoke and maternal conditions, including gestational hypertension (76) and gestational diabetes (69), but more studies are needed to confirm these findings. Underlying mechanisms are attributable to systemic inflammation, placental inflammation, reactive oxygen species, and toxic effects on organ developmental trajectories (62, 76, 77).

#### Hurricanes and floods

Hurricanes are fueled by warm oceanic temperatures, which have intensified as a direct result of human-caused global warming (78). In fact, modern hurricanes in the Atlantic have been significantly more intense relative to projections without the influence of human-driven warming (79).

Hurricanes and floods have been associated with preterm birth in Australia (80) and in the United States, especially among socially disadvantaged populations (81). Separately, floods have been associated with low birthweight and severe maternal morbidity in South Carolina (82). Researchers have documented the impact of hurricanes in Puerto Rico on increased instances of postpartum depression and exposure to environmental contaminants, including various metals and the phthalate congener DEHPTP, used in plastics to make them pliable (83, 84). The mechanisms underlying these outcomes continues to be investigated but may be explained by established risk of stress-related changes to maternal-fetal endocrine and immune processes (85), as well as interruptions in prenatal care (86) and food security (87) experienced in the aftermath of natural disasters.

Intensifying floods also pose a significant risk to reproductive health, as they are projected to increase by more than 60% worldwide (88). Rushing water mobilizes pollutants, which can contaminate water supplies and compromise sanitation, leading to the outbreak of waterborne diseases and more breeding of mosquitoes and pathogens (89). Aside from the direct impact of the pollutants, the weather events themselves can indirectly affect reproductive health. For example, flooding can lead to food shortages and limited supplies, putting strain on pregnant women and feeding mothers, adversely affecting maternal and child health, resulting in low birthweight and increased susceptibility to infection (90). Natural disasters can also disrupt maternal and pediatric health services, leading to delayed diagnosis and treatment of health conditions (91).

## Subsequent Impacts in a Changing Climate and Reproductive Health

### Downstream and Indirect Impacts of Climate Change

The frequency and intensity of weather-related disasters have escalated significantly in recent years, a pattern firmly attributed to the impacts of climate change. According to the Atlas of Mortality and Economic Losses from Weather, Climate, and Water Extremes (1970-2019), published by the World Health Organization, the incidence of extreme weather events such as heatwaves and flooding has increased 5-fold over the past 6 decades, with substantial evidence linking these surges to human-induced climate change (92). The distribution of climate risk varies geographically and socioeconomically. According to the 2023 IPCC report published in 2023, the most vulnerable populations at risk for extreme heat reside in sub-Saharan Africa and Southeast Asia, which are disproportionately susceptible to droughts associated with increasing global greenhouse gas emissions (4). Nations in the Caribbean, which experience high levels of poverty and maternal morbidity, are projected to experience rising levels of destruction secondary to intensifying hurricanes (93). Meanwhile, Southeast Asia has experienced the heaviest burden of deaths attributed to floods over the past 30 years (94).

Across the globe, socioeconomic disparities also increase or compound climate risk experienced by low-income communities. Even in affluent countries, poverty mediates exposure to natural disasters, pollution, and access to food resources (24). Climate change exacerbates existing poor health outcomes by interfering in the resiliency of impoverished communities during extreme climate events. This often arises because impoverished communities are forced to live in less climate-protected areas and have higher levels of chronic disease (95). For example, poor and Black neighborhoods in New Orleans faced a disproportionate level of suffering and death in the aftermath of Hurricane Katrina (96), the costliest natural disaster in United States history (97).

Downstream effects of climate change, including the reproductive ramifications of drought, food scarcity, infectious disease, and exposure to air pollution, lead to poor maternal and fetal health outcomes via various mechanisms including endocrine disruption, stress, and immune-related physiology, as we present next.

### Air Pollution

Climate change and air pollution are closely interrelated, in that some sources of air pollution, such as traffic and industrial emissions, contribute to climate change, whereas others, like intensifying wildfires, are consequences of climate change. Both are commonly assessed by measuring concentrations of PM_2.5_—particulate matter with a diameter of 2.5 µm or smaller—which serves as a general biomarker of air pollution and is a well-established risk factor for numerous adverse health outcomes including impaired fertility and pregnancy complications worldwide (8).

In 2019, 99% of the world's population lived in areas where air quality failed to meet guidelines set by the World Health Organization aimed at preventing respiratory disease and other adverse health outcomes associated with air pollution (98). Importantly, ∼3 billion people are also exposed to indoor residential air pollutants in their homes, particularly from wood-fired stoves that contribute to approximately 2 million deaths per year (99). Emissions such as PM_2.5_, PM_10_, and polycyclic aromatic hydrocarbons (PAH) have shown consistent association with increased risks of several poor pregnancy outcomes, including altered birthweight, preterm birth, and birth defects that are plausibly causal (11, 100, 101). Gestational exposures to elevated levels of these air pollutants are suspected to lead to inflammatory responses that negatively affect fetal growth and development (102). Further, exposures to air pollutants such as particulate matter may also impact endocrine signaling critical for both male and female reproductive health (103, 104).

The impacts of air pollution on human health are wide-reaching, in part because pollutants like PM can diffuse through human tissue and accumulate in tissues and organs (105). PM_2.5_ and smaller ultrafine particles cross biological barriers like the blood-testis barrier, allowing accumulation in reproductive organs (106). In fact, black carbon and PM_2.5_ can localize within both cellular and extracellular components, including germ cells, seminal plasma, ovarian follicular fluid, the placenta (107), as well as the developing embryo or fetus (106). In rodents, PM_2.5_ impairs placental development by inducing inflammation, hypercoagulability, and vascular thrombosis potentially compromising delivery of oxygen and nutrients (108). PM_2.5_ in humans has also been associated with decreased placental chorionic disk area (109), an area crucial for fetal growth and development.

Increased air pollution exposure is associated with adverse pregnancy outcomes (eg, spontaneous miscarriage) and maternal complications, such as preeclampsia and gestational hypertension, which pose severe risks to both maternal and fetal health (110). Such maternal conditions predispose to preterm birth and low birthweight, which are linked to an increased risk of health risks later in life (111). Moreover, exposure to air pollution is associated with an increased infertility and miscarriage, with fine PM_2.5_ and nitrogen dioxide (NO_2_) being particularly detrimental to pregnancy outcomes (11). Neonatal and childhood exposures to ambient pollution have also been linked to negative health outcomes such as perinatal mortality, sudden infant death syndrome, neurobehavioral disorders and neural tube/heart defects (112). Additionally, these risks are compounded for populations living in urban and industrial areas, where pollution levels tend to be highest due to dense traffic and industrial emissions.

Anthropologic activity, including industrial pollution, is expected to exacerbate wildfires, especially in settlements and infrastructures built with flammable vegetation, which has the potential to cause these carbon stores to contribute to the greenhouse effect via release of PM. This poses increased risk of preterm birth, especially when exposure occurs within the second trimester (69), attributable to a stress response that results in premature onset of labor (70).

Air pollutants contribute to reproductive toxicity and adverse health outcomes through a range of biological mechanisms. Major air pollutants (eg, PM, NO_2_) and common ambient chemical constituents (eg, PAHs, toxic metals), induce oxidative stress through the generation of reactive oxygen species and inflammation. These biological effects are associated with a variety of adverse outcomes, including altered ovarian function, reduced sperm quality, and disrupted placental development and function, driven by both direct or indirect effects on reproductive tissues (113-118). Growing evidence also highlights the impact of air pollutants on maternal and fetal immune function, including disruptions in cytokine signaling, elevated systemic inflammation, and altered immune cell profiles (119, 120). These immunological perturbations have been implicated in adverse pregnancy outcomes such as preeclampsia, preterm birth, and impaired placental function as well as long-term risks to maternal and children's health (115-118).

Additionally, several air pollutants can directly induce DNA damage or impair DNA repair mechanisms, with some, such as benzo[a]pyrene, a mutagenic PAH, exhibiting genotoxic effects that may further impair reproductive function. In polluted environments, these effects may be especially pronounced in individuals in occupations with high exposure to exhaust fumes (121).

Air pollutants can also induce epigenetic alterations, including changes in DNA methylation and histone modifications, which may disrupt gene expression critical for reproduction, including placental growth, fetal development, and birthweight (122). Prenatal exposure to PM and other air pollutants are associated with altered DNA promoter methylation of steroidogenic enzyme genes and key regulators of fetal growth (123, 124). Furthermore, ambient pollution is linked to epigenomic changes and impaired mitochondrial function in placental cells, suggesting reduced functionality (125).

It is also important to underscore that communities exposed to air pollution are not affected equally, and often disenfranchised populations experience disproportionate amounts of environmental injustice (126). For example, historically marginalized communities like Native Americans and Alaska Native populations are statistically more likely to experience disproportionate PM_2.5_ exposure (127). Additionally, although only 6% of White neighborhoods in fire-prone Southern California are employed in outdoor occupations like construction, delivery, transportation, and agriculture, 17% of Latino residents work in these sectors (128). In turn, these communities experience close to double the exposure to diesel and PM_2.5_ pollution compared to White neighborhoods, compounding existing health disparities. More broadly, work disruptions posed by wildfires affect low-income workers' ability to get paid, potentially disincentivizing them from staying at home when air quality is poor and widens the exposure gap across socioeconomic strata.

Endocrine disrupting chemicals are found in fossil fuels contributing to climate change, although they are rarely considered in that context, but rather through the lens of the individual, occupational, and community exposures due to personal and industrial uses and pollution. Their effects on reproductive health have been extensively reviewed (7, 8, 17, 20, 21, 48, 111, 129, 130), and next we contextualize them in the setting of climate change and its downstream effectors.

### Endocrine Disrupting Chemicals

EDCs are defined as chemicals that can alter, induce, or antagonize hormone synthesis, signaling transduction, metabolism, or transportation via a variety of mechanisms (129). With these characteristics, the reproductive systems in males and females that respond to and/or synthesize a variety of steroid hormones, are prime targets of disruption with major impacts on reproductive and developmental competence, outcomes, and consequent longer term health compromise. EDCs comprise a diverse group of compounds, including pesticides/insecticides (eg, DDT, organochlorines), petrochemicals, and select pharmaceutical agents (eg, synthetic estrogens), phenolic compounds, such as bisphenol A and phthalates (plasticizers and personal care products), and polychlorinated biphenyls, flame retardants, and components of diesel fuel exhaust (17, 111, 131, 132). Routes of exposures to individuals and communities transdermally, transplacentally, and via ingestion and inhalation (by transport or diffusion with potential to affect placental function and fetal growth and development) (111, 133-135). The temperature increase worldwide has also contributed to an increase in natural disasters that can increase exposures to contaminants that impact reproductive health. For example, natural disasters such as hurricanes and flooding displace these compounds into water and the environment, increasing population exposures (136).

EDCs affect reproductive tract development, physiology, function, and fertility via multiple mechanisms. These include disruption of estrogen, androgen, progesterone, and thyroid hormone signaling, the hypothalamic-pituitary-gonadal axes, gonadal and reproductive tract development and functions, and placental development, growth, cell proliferation, and differentiation (e.g., syncytium formation, cytotrophoblast differentiation, vasculogenesis, steroidogenesis, immune tolerance of the conceptus), and can have impacts on metabolism and systemic immune processes (19, 20, 111, 129, 130, 134, 137). In addition, EDCs commonly promote local and systemic inflammation that contributes to poor reproductive outcomes (111, 130). Specific EDCs can affect molecular targets (e.g., COX2, NFkB, PGE2), cell proliferation, differentiation, and invasion, transporter expression, kinase and other signaling pathways, endoplasmic reticulum stress, apoptosis, oxidative stress, steroidogenesis, cytokine signaling, hormonal transport and synthesis, clearance, and immune cell dysfunction (19, 21, 111, 130, 134). These disruptions can impair the development and function of reproductive organs and contribute to infertility, poor pregnancy outcomes, and/or pregnancy complications. Not all EDCs share these mechanisms, but notably, exposures to mixtures of chemicals are nearly ubiquitous and together can create a greater risk of adverse health consequences (138). Thus, multiple processes can be affected with mixture exposures—competitively, synergistically, or neutrally. Authoritative bodies have identified that chemical exposures can in combination more greatly increase the risk of adverse health outcomes and should be addressed accordingly.

Fecundity and fertility have decreased over the past 40 years while rates of miscarriage and preterm birth have increased (6). Declining semen quality and sperm count have been well-documented (139, 140), and there is ample evidence that EDCs are impacting these aspects of reproductive health, although to what extent is uncertain (141). Female fertility is also impacted, with EDCs such as bisphenol A adversely impacting ovarian reserve (142). EDC exposure can also advance reproductive aging, as demonstrated by women exposed to higher levels of environmental pollutants and EDCs experience menopause approximately 1.9 to 3.8 years earlier than those with lower levels of exposure (89). This early onset of menopause not only affects fertility but also increases the risk of associated health conditions such as osteoporosis and cardiovascular disease. Furthermore, EDC exposure has been shown to exacerbate menopausal symptoms, including hot flashes, night sweats, and mood disturbances, thus prolonging and intensifying the symptom burden for affected individuals (89). Per the National Academy of Sciences guidelines, clinical follow-up and monitoring of EDCs may be merited for patients with elevated exposure to provide them with additional health screening (138).

With regard to male fertility, rising ambient temperatures and exposure to EDCs are associated with decreased sperm production, quality, and motility (143, 144). In vivo exposure to the PAH benzo[a]pyrene has been associated with altered steroidogenesis and reduced testosterone production in rat Leydig cells (145). Similarly, in utero exposure to another PAH, phenanthrene, causes a transgenerational decline in Leydig cells, decreased serum testosterone, impaired spermatogenesis, and reduced male fertility in both F1 and F2 generations (146).

Gestational exposure to the heavy metal cadmium lowers progesterone levels in maternal sera, placentae and amniotic fluid, disrupts the expression of key steroidogenic enzymes, and induces protein kinase-mediated placental mitophagy, contributing to fetal growth restriction (147).

Early-life exposure of female mice to the persistent dioxin 2,3,7,8-tetrachlorodibenzo-p-dioxin reduces uterine progesterone responsiveness and the expression of progesterone-regulated proteins critical for establishment and maintenance of pregnancy, resulting in subfertility, spontaneous preterm birth, and endometriosis-like histological and functional phenotypes that persist in F3-F4 generations in the absence of chemical exposure (137, 148).

EDCs can be lipophilic allowing them to bioaccumulate in the adipose tissue (111). Once released, these substances can enter food sources such as dairy, poultry, seafood, and livestock, which are then consumed by humans (111). Some EDCs also persist within tissue for extended periods, posing ongoing health risks even after initial exposure (111). The per- and polyfluoroalkyl substances (PFAS), labeled as “forever chemicals,” have been detected in the blood of both humans and wildlife worldwide, underscoring their pervasiveness in the global food chain (149). PFAS have been identified at increasing levels in the marine environment, with bioaccumulation throughout the food chain, from phytoplankton (150) to marine animals, drinking water and foods (151). They have been identified in every human analytically tested for their presence and increase the risk of inflammatory bowel and thyroid disease, reduced fertility, testicular cancer, and pregnancy-related complications (138). Climate change exacerbates exposure to PFAS via storms, flooding and the spread of pollutants into surface waters and soils, making them more ubiquitous (152). The presence of EDCs across these varied biological matrices underscores the pervasiveness of human exposure and indicates that the fetuses are at risk. This persistence and widespread distribution in the body suggest a cumulative exposure over time, heightening the potential for long-term endocrine and reproductive disruption associated with these chemicals.

#### Drought and food availability

Crop shortages associated with climate change can lead to food insecurity and threaten the livelihoods of families in areas most vulnerable to extreme weather. The Council on Foreign Relations projects that more than 143 million people across vulnerable regions in Asia, Latin America, and sub-Saharan Africa could be displaced by extreme climate conditions and become climate refugees by 2050 (153). Climate migration in turn poses geopolitical and humanitarian challenges as receiving countries face pressure from constituents to control border crossings (154).

Droughts affect both reproductive health and exacerbate existing health disparities. As of 2020, Chad, Nigeria, and South Sudan had the highest rates of maternal mortality (155). Sub-Saharan nations are also expected to bear the burden of extreme droughts and floods as weather systems intensify throughout the African continent and likely compound poor reproductive health outcomes. Droughts have hit historic levels in Northern Africa, which led to a decreased harvest in both 2022 and 2023 (156). Droughts have been associated with lower birthweight in Nepal (157) and the cascading impacts of droughts (and other climate change factors) on food security is associated with poor maternal-fetal outcomes, such as vitamin deficiencies, maternal mortality, and infant malnutrition (158). These outcomes have persisted in countries such as Uganda (159), despite improving antenatal care. Crop shortages and associated malnutrition pose unique risks to pregnancy and developing fetuses in these regions, as nutritional status serves a critical role in resilience against heat-associated maternal stress and fetal growth restriction (160).

#### Infectious disease (vector-borne)

Rising global temperatures and shifts in weather patterns, including prolonged rainy seasons, create conditions that facilitate the proliferation of vectors like mosquitoes and pathogens, which thrive in warmer, wetter environments. These climate-driven changes have significant public health implications, particularly for pregnant individuals and fetal health. These infectious indirect effects of climate change on reproductive health are numerous and often hard to capture. Increases in obstetric complications are associated with climate stressors, such as communicable disease and migration patterns. Expanding mosquito populations in the aftermath of extreme weather events raise the risk of arboviral infections, such as Zika and dengue, during pregnancy, heightening the potential for severe outcomes, including abnormal fetal development and congenital conditions like microcephaly (89). The number of people living in suitable transmission temperatures for Zika is expected to increase to 1.3 billion by 2050 (161) because of warming across the globe.

## Mitigation Strategies

The impacts of climate change disproportionately affect women, particularly those of lower socioeconomic status, pregnant women and their developing fetuses, the elderly, the disabled, and young children (5, 162). Socioeconomic factors including housing (163), access to clean air and water, and health care, can further exacerbate risks associated with climate change.

Mitigating the health and reproductive impacts of climate change will require a coordinated effort across governmental, industrial, and regulatory bodies. In particular, government policies addressing climate change are essential for equitable transition away from the primary causes of climate change, as voluntary programs do not lead to lasting and sustained changes. Governmental policies to address climate change should integrate adaptational strategies to protect communities most vulnerable to extreme weather and address the underlying drivers of climate change, including greenhouse gas emissions and burning of fossil fuels. The IPCC identifies 1.5 °C of warming above preindustrial levels as a threshold to stave off the worst impacts of climate change, including significant decline in marine life, sea level rise, wildfire exacerbation, and extreme heat-related deaths (4). On the current global trajectory, warming is projected to reach 3 °C by 2100, with warming continuing thereafter, underscoring the urgency required to galvanize international stakeholders.

### Government Actions to Reduce Climate Change Causing Pollution

Legislation targeting greenhouse gas emissions serves as one of the most powerful tools to mitigate the impacts of climate change. Many policies have focused on carbon dioxide emissions, but policies need to expand to also address methane gas which has 28 times the global warming potential as carbon (164). According to the Climate and Clean Air Coalition, global burning of methane, black carbon, and fuel must decrease by 30% from 2020 levels by 2050 to achieve the goal of not exceeding 1.5 °C of warming (165). Cutting the consumption of these fossil fuels will require global adoption of renewable energy sources, and significant transformation of energy production across nations and industries. Renewables should comprise 75% to 80% of world energy sources to meet IPPC goals (166). Because of government policies and investments, the price of solar and onshore wind electricity declined by 89% and 70%, respectively, in the past decade alone (167). Policies like the 2022 Inflation Reduction Act are projected to reduce emissions by 40% from 2005 levels by facilitating the transition to renewables. In California, cap and trade strategies can also play a pivotal role in reducing emissions, having already decreased statewide emissions by 5.3% from 2013 through 2017 (168). Although this reduction represents much needed progress, overall emissions reduction and supplemental technologies like carbon capture throughout the energy sector are required to meet the Paris Agreement milestones by 2030.

### The Role of Transportation Innovation in Curbing Emissions

Although transportation accounts for 14% of greenhouse gas emissions, 45.1% of transportation-related pollution stems from passenger vehicles, presenting an opportunity to make inroads with the electric vehicle (EV) market. In fact, the Clean Air Act and recently amended Environmental Protection Agency standards have led to the explosion of EV sales in the United States in recent years (169). In some countries, EVs have even started to dominate the automobile market (170). From 2022 to 2023 alone, the share of new electric car sales increased globally by 35% (170). Policies tackling emissions should continue to incentivize EV technology, while at the same time lowering emissions from transportation vehicles toward a goal of zero emissions. Furthermore, revitalizing broader transportation infrastructure can mitigate fuel consumption altogether. The implementation of bike lanes and active transportation infrastructure significantly reduces fossil fuel consumption per capita (171). Investment in low-emission modes of transportation, such as cycling and rail infrastructure, provides an opportunity to make big dents in overall emissions.

### Practical Approaches and Protective Measures

Although cutting carbon emissions remains an important priority, it is also critical to implement interventions to protect communities against climate change in the short term. Protections against different exposures and climate impacts vary, but efforts should be made to reduce the disparities of these exposures to prioritize environmental justice.

#### Housing protections

Air pollution from fossil fuels disproportionately impacts lower-income communities globally, especially in urban areas (172). Outdated housing infrastructure often lacks adequate filtration systems and may include older stoves and kitchen appliances that contribute to indoor air pollution. In fact, low-income homes may have 4.5 times the median of PM2.5 indoors compared to outdoors during wildfire season (173). Revitalizing housing infrastructure—especially in poor populations—to include filtration systems and nonpolluting stoves and ovens, would serve to reduce home energy emissions while also protecting pollution from infiltrating inside. In fact, a study done at Columbia University in 2021 to 2022 showed that replacing gas stoves with induction or electric stoves resulted in a 56% reduction in household NO_2_ concentrations (174). New York became the first state to ban newly constructed buildings from using natural gas heating and appliances by 2026 (175). Although curbing emissions in new buildings helps address sources of indoor pollution, investment in revitalizing existing buildings should take priority as well. Gas stoves are still pervasive in the United States, present in over one third of homes (176), including in sections of public and government-subsidized housing nationwide. Given that these units disproportionately house low-income residents with existing health disparities (177), efforts to mitigate exposure to indoor pollution must extend to the public housing authority.

#### Workplace protections

Climate change also poses occupational hazards for both female and male reproductive health, as well as pregnancy of agricultural and industrial workers. These laborers experience unique risks, including exposure to extreme heat, pesticides, and poor air quality during wildfire seasons. Pesticides are associated with fetal growth restriction, low birthweight and premature birth (178). In fact, pregnant agricultural workers are more likely to give birth to babies significantly smaller than women in other industries (179). Qualitative studies have also underscored that agricultural workers, especially migrants, feel they lack control and information regarding their exposures (180). In the United States, absence of a universal health care system and guaranteed employee benefits may also disincentivize workers from staying indoors or taking a day off when air quality is poor. Migrant workers have increased rates of respiratory illness related to occupational dust and pollution exposure (181). Data show that improving workplace protections by reducing workplace exposures benefits workers and could help curb poor health outcomes experienced by laborers in this sector. In California, state law provides special protections to pregnant agricultural workers and guarantees their right for workplace accommodations, regardless of immigration status (182). The Occupational Safety and Health Administration also has clear guidelines regarding heat parameters that trigger heat stress guidelines and prevention protocols (183). Enforcing these protections and expanding them to include protections against EDCs and volatile chemicals has not yet been achieved in the workplace but it is imperative for the reproductive health of laborers in this crucial sector. Occupational health experts can play a critical role in advocating for the standardization of protections across agricultural and biomedical fields to lower exposure to EDCs.

#### Role of education and advocacy

Last, campaigns to increase community awareness around climate change are vital to integrating and standardizing environmental protections, as well as galvanizing social and legislative change. Health care professionals, community leaders, policymakers, and researchers can each play a role in educating the public and advocating for safeguards around hazardous exposures. Medical societies nationwide and across the globe have a shared responsibility to maintain abreast of the latest research findings regarding the impact of climate and environmental exposures on reproductive health and take proactive steps to educate the public, health policymakers, research and health care professionals. These include the Endocrine Society, International Federation of Gynecology and Obstetrics, World Health Organization, Medical Society Consortium on Climate and Health, American Academy of Pediatrics, American Congress of Obstetricians and Gynecologists, International Society for Social Pediatrics and Child Health, Association of Women's Health, Obstetric & Neonatal Nurses, Partnership for Maternal, Newborn and Child Health, and Global Climate and Health Alliance, among many others. Although much of the public health discourse understandably focuses on maternal health disparities, coordinated efforts should also be made to educate nonpregnant women and men on the environmental impacts of EDCs and pollution on their reproductive health.

## Conclusion

Climate change will continue to exert effects on reproductive health via air pollution, extreme weather, and increased exposure to contaminants unless stakeholders and regulatory bodies take significant measures to curb their circulation. Although existing studies strive to explore the mechanisms by which these changes to reproductive health outcomes occur, more research and advocacy warrants focusing on strategies to protect vulnerable populations, such as reproductive age persons, pregnant individuals, and low-income communities, from these exposures. Future research would benefit from using recent advancements in simulation models, exposure trackers and geometric exposure estimates to investigate areas of study–such as causal mechanisms on climate on reproductive and maternal, fetal, and children's health—that have traditionally been harder to disaggregate (184). Coordination across scientific, private sector, and legislative bodies to detect and minimize exposures to pollutants and contaminants would have the inherent benefit of reducing reproductive health disparities.

## Current Affiliation

Sanika Pelnekar current affiliation is with the John Sealy School of Medicine at The University of Texas Medical Branch, Galveston, TX, USA.

## Abbreviations

EDC: endocrine disrupting chemical
EV: electric vehicle
NO_2_: nitrogen dioxide
PAH: polycyclic aromatic hydrocarbon
PFAS: per- and polyfluoroalkyl substances
PM: particulate matter
